# Changes in antioxidant and antibacterial activities as well as phytochemical constituents associated with ginger storage and polyphenol oxidase activity

**DOI:** 10.1186/s12906-016-1352-1

**Published:** 2016-09-29

**Authors:** Ali Ghasemzadeh, Hawa Z. E. Jaafar, Asmah Rahmat

**Affiliations:** 1 Department of Crop Science, Faculty of Agriculture, Universiti Putra Malaysia, 43400 Serdang, Selangor Malaysia; 2 Department of Nutrition & Dietetics, Faculty of Medicine & Health Sciences, Universiti Putra Malaysia, 43400 Serdang, Selangor Malaysia

**Keywords:** Antibacterial activity, Antioxidant activity, Flavonoids, Ginger, Phenolic acids, Storage time, Storage temperature

## Abstract

**Background:**

Herbal materials should be stored at optimal conditions in order to retain their nutritional quality. Proper storage has a significant impact on the quality of the herbs and spices.

**Methods:**

The effects of storage temperature (5 and 15 °C) and time (4 and 8 months) on the phytochemical constituents associated with the antioxidant and antibacterial activities of ginger varieties (Halia bentong and Halia bara) were evaluated to determine the optimal storage conditions for ginger rhizomes. Total flavonoid content (TFC) and Total phenolic content (TPC) were measured using the spectrophotometric method. Individual phenolic acids and flavonoids, 6-gingerol and 6-shogaol were identified by ultra-high performance liquid chromatography. Ferric reducing antioxidant potential (FRAP) and 1,1-diphenyl-2-picrylhydrazyl (DPPH) assays were used for evaluation of antioxidant activities. An antibacterial property of ginger varieties was evaluated using well diffusion method.

**Results:**

Dry matter, TPC, TFC and individual phenolics and flavonoids content, 6-gingerol and 6-shogaol content noticeably decreased at 5 and 15 °C during the storage times from 4 to 8 months. Highest content of flavonoids, phenolic acids, 6-gingerol, and 6-shogaol was observed in fresh samples followed by rhizomes stored at 5 °C for 4 months. Storage at 15 °C for 4 months reduced the phytochemical content significantly. Cinnamic acid and tannic acid were not detected in those variety stored at 15 °C for 4 and 8 months. Polyphenol oxidase (PPO) activity was associated significantly with storage time and temperature. Highest and lowest PPO activity was observed in stored and fresh rhizomes respectively. Antioxidant and antimicrobial activities gradually declined with the increase of storage temperature (from 5 to 15 °C) and duration (from 4 to 8 months) in both the varieties. Freshly harvested Halia bara variety had higher antioxidant and antibacterial activity compared to the Halia bentong variety.

**Conclusions:**

Halia bara exhibited valuable phytochemical content and antioxidant and antibacterial activities at higher levels compared to that exhibited by Halia bentong rhizomes. In general, storage of Malaysian ginger varieties at temperature of 5 °C is recommended and the storage time should be not more than 4 months. This storage condition will provide greater stability to the concentration of the phytochemical constituents more similar to the fresh material.

## Background

As a good source of bioactive compounds, medicinal plants/herbs have long been utilized both in modern and traditional medicineas. They are also used as chemical antecedents and pharmaceutical intermediates for synthetic drugs [1]. Today, avaluable line of study in the medical industry is appraising phytochemicals to ascertain whether they have boilogical activities with potential benefits to human health. Zingiberaceae plants have received much attention, since they produce various compounds that are useful in food as spices and herbs, seasoning agents and flavouring, and in the medicinal and cosmetics industries as antimicrobial and antioxidant agents. Ginger (*Zingiber officinale* Roscoe), locally known as Halia in Malaysia, is a widely used spice in the world. Due to its worldwide appeal, ginger use has spread to most tropical and subtropical countries from China and India, where ginger cultivation has been prevalent, possibly since prehistoric times [2]. In the ancient times, ginger played significant role in primary health care in India and China and was highly valued for its medicinal properties. Ginger contains a variety of pungent and biologically active compounds such as phenolics, flavonoids, gingerol, shogaol and zingerone [3]. From among the identified components, gingerol was found to be the most abundant bioactive compound in ginger with numerous pharmacological effects including antioxidant, analgesic, anticancer, antipyretic and, anti-inflammatory properties [4, 5]. Many factors such as environment, and cultivation conditions such as daily light intensity and temperature, type and range of fertilizer application, production time, watering/irrigation and plant age are considered to signifycantly affect the levels of health-promoting compounds in herbs and crops [6, 7]. Herbal materials must be stored under specified conditions in order to retain their nutritional quality and avoid contamination and deterioration. Proper storage has a significant impact on the quality of the produce in general [8].

It was recommended that fresh/dried ginger rhizomes, splits or slices must be keep and stored in a cool environment with temperature ranging 10–15 °C [9]. Ginger, when stored at room temperature (23–26 °C) for three months lost up to 20 % of its oleoresin (dry weight), and the content of (6)-gingerol also decreased [10]. Therefore, if cold storage is not available, it is recommended to extract or distil dried ginger expeditiously into the final product, oil or oleoresin. The importance of dry storage for dried ginger destined for distillation cannot be over emphasised because of the risk of mycotoxins from mould getting co-distilled with the essential oil [11]. However, cold storage may not always be available in the production areas. In a study on Hawaiian ginger, rhizomes stored at 12.5 °C showed stable quality (oil, sugar, and phenol content) for 28 weeks [12]. In comparison, storage at 22 °C shortened the commercialisation window to 20 weeks due to excessive water and fibre content loss. Ginger is also prone to chilling injures when stored below 7 °C which resulted in pitting and sunken lesions on the surface, softening, browning, and decay [9]. Since it is difficult and expensive to preserve the ideal storage conditions for ginger rhizomes, farmers tend to store rhizomes in underground tunnels, where the maintenance of optimum humidity and temperature control are impossible. This results in spoilage and the ginger roots sprout after a few months. Developing a method for the long-term storage of harvested ginger roots, without a loss in the quality, is very important to ginger growers, ginger-processing personnel, and its perennial consumers. It appears that storing at 5–15 °C could be a suitable method to preserve the quality of the ginger roots for a long period, but not much data were reported in this area. There is also no published data currently available on the effect of storage time and temperature on phytochemical constituents and pharmaceutical quality of Malaysian ginger varieties. The aim of this study was to evaluate the effect of storage time and temperature on phytochemical constituents (TPC, TFC, and individual phenolics and flavonoids, 6-gingerol and 6-shogaol) and changes in antioxidant and antibacterial activities of two of the Malaysian ginger varieties, Halia bentong and Halia bara.

## Methods

### Plant sampling

The rhizomes of Halia bentong and Halia bara were collected from a village in Bentong, Pahang, Malaysia. Rhizomes were washed with pure water and socked for 30 min in a solution of Mancozeb (0.3%). The rhizomes were cut at 3–5 cm in size containing 2 to 3 buds and were germinated for two weeks in small pots containing peat moss (1 kg) under glasshouse condition. After two weeks, seedlings with 2 to 3 leaves were transplanted into polyethylene bag (6 L) filled with soilless mixture (burnt rice husk and coco peat, 1:1). The mean daily temperature 30 °C, mean relative humidity 80 % and highest irradiance level at 1650 μmol/m^2^/s and whilst minimum at 44 μmol/m^2^/s. Harvesting was done at 9 month after plantation. Rhizome, leaf and stem were separated and washed with pure water. Samples for storage treatments were cured at 29 °C and 85 % relative humidity for 7 days, and then subsequently stored for 4 and 8 month in the dark at 5 and 15 °C and 80–85 % humidity. Ginger seedlings (whole plant) were sent to Institute of Bioscience (IBS), Universiti Putra Malaysia and were identified as *Zingiber officinale* Roscoe and *Zingiber officinale* Roscoe var. *rubrum* Theilade. The voucher specimen were identified and deposited at IBS Herbarium.

### Estimation of dry matter content

Rhizome samples were taken for analysis immediately after harvest (fresh) and after 4 and 8 months of storage. Before analysis, freshly harvested and stored ginger samples were washed, towel dried, peeled, cubed (2 cm), weighed and immediately frozen at −80 °C before lyophilization. The freeze-dried material was weighed and the dry matter content was estimated by difference in weight. Lyophilized rhizomes were subsequently powdered by grinder and stored at −80 ˚C until extraction. All analyses were conducted in triplicate.

### Extraction

Lyophilized rhizome samples (5 g) were extracted with ethanol (100 mL). Extraction was done using refluxed at 65 °C for 35 min. Then, solutions were cooled at room temperature and filtered through Whatman filter paper (No. 1). Excess solvent was removed by evaporation using rotary evaporator. Residue was freeze dried and was kept at −20 °C for future analysis.

### Total phenolic content

The total phenolics content of ginger extracts was determined using the Folin–Ciocalteu reagent method with slight modification [13]. In brief, 1 mL of rhizome extracts (1 mg/mL in ethanol) were mixed with 9 mL of distilled water. For the assay, 1 mL of 10-fold diluted Folin-Ciocalteu reagent was added and shaken gently. After 10 min, 1 mL Na_2_CO_3_ (7 %) solution was added to the mixtures and total volume was brought to 25 mL by adding distilled water followed by incubation for 90 min in the dark. The absorbance was measured against a blank at 760 nm using UV–visible spectrophotometer. Gallic acid was used to prepare a calibration curve (*R*^*2*^ = 0.988). Results were expressed as milligram gallic acid equivalents (GAE)/g dry material (DM).

### Total flavonoid content

Total flavonoid content was measured using aluminium chloride colorimetric method. In brief, 50 μL of rhizome crude extracts (1 mg/mL ethanol) were mixed with 4 mL of distilled water and 0.3 mL NaNO_2_solution (5 %); after 6 min incubation at 25 °C, 0.3 % AlCl_3_ solution was added. The mixture was mixed well, and allowed to stand for another 60 min. Absorbance was measured at 510 nm against a blank using UV–visible spectrophotometer. Quercetin standard was used to prepare a calibration curve(*R*^*2*^=0.991). Results were expressed as milligram quercetin equivalents (QE)/g DM [1].

### Identification and separation of flavonoids and phenolic acids

The chromatographic separation of the flavonoids and phenolic acids was performed using a Ultra-high performance liquid chromatography (Agilent, Model 1200). The flavonoids and phenolic acids were separated on a Agilent C18 (5 μm of particle size, 4.6 × 250 mm) reversed-phase column by gradient elution using: 0.03 M orthophosphoric acid (A) and methanol HPLC grade (B). The gradient profile was: 0 min 40 % B, 10 min 100 % B, 15 min 100 % B, and 20 min 40 % B. The detector wavelengths were set at 280 and 360 nm. The flow rate and injection volume was 1 mL/min and10 μL, respectively. Column temperature was set at 35 °C. Identification of the compounds was achieved by comparison of retention times with standards, UV spectra and UV absorbance ratios after co-injection of samples and standards.

### UHPLC analysis of 6-gingerol and 6-shogaol

The UHPLC system (Agilent, Model 1200) was used. The 6-gingerol and 6-shogaol were separated on Agilent C18 (4.6 × 250 mm, 5 μm) reversed-phase column by gradient elution using: (A) water and (B) acetonitril. The detector wavelengths were set at 280 nm. The flow rate and injection volume was 1 mL/min and 20 μL, respectively. Identification of the compounds was achieved by comparison of retention times with standards (6-gingerol and 6-shogaol), UV spectra and UV absorbance ratios after co-injection of samples and standards. System suitability requirements: Perform at least five replicate injections of 6-gingerol and 6-shogaol. The requirements of the system suitability parametersare: (1) Symmetry factor is not mo.re than 1.5 (2) Percentage of relative standard deviation of the retention time for 6-gingerol and 6-shogaol standards is not more than 2.0 %.

### Evaluation of antioxidant activity

#### 1,1-Diphenyl-2-picrylhydrazyl (DPPH) assay

A 100 μM methanolic solution of DPPH was freshly prepared (eliminated from light) and 3 mL of this solution was mixed gently with 3 mL of ginger extracts. All solutions were incubated at 25 °C for 30 min in darkness. The absorbance of the test and standard solutions was recorded against blank (methanol and DPPH solution without sample) at 517 nm using UV–visible spectrophotometer. Gallic acid and quercetin were used as a standard control [14]. The percent of inhibition was calculated using the following formula:

1 $$ \%\mathrm{inhibition}=\left[\left(\mathrm{A}{\textstyle \hbox{-}}\mathrm{B}\right)/\mathrm{A}\Big)\right]\times 100 $$

where, A is absorbance of control; B is absorbance of sample.

#### Ferric Reducing Antioxidant Potential (FRAP) assay

FRAP reagent: 2.5 mL FeCl_3_ (20 mmol/L); 2.5 mL (10 mmol/L) 2,4,6-tripyridyl-S-triazine (TPTZ) and 25 mL acetate buffer (PH = 3.6, 0.3 mol/L) was mixed gently and incubated in water bath at 37 °C for 20 min (eliminated from light). Ginger extracts (0.2 mL) were dissolved in 2.0 mL of the FRAP reagent and made up to 10 mL. Mixture was incubated at 25 °C for 30 min in the water bath. The absorbance of the solution (blue colour) was read against blank (acetate buffer) at 593 nm using UV–visible spectrophotometer. A standard cur.ve was prepared using concentrations of 100–1000 mM of FeSO_4_ × 7H_2_O. The results are expressed in μM of Fe (II)/g DM [15].

### Polyphenol oxidase activity (PPO)

A total of 800 μL of catechol (50 mM) was mixed with 50 mM phosphate buffer (pH 6.5), then 200 μL of enzyme extract was mixed with this reaction solution and incubated at 37 °C for 2 h. A sample of 1 mL of substrate solution was used as a control. Absorbance of solutions were measured at 475 nm using UV–visible spectrophotometer [16]. Differences in the absorbance (before and after 60 sec) of the sample and control was calculated as PPO activity and expressed as Δ475/min g DM.

### Antibacterial activity

#### Antibacterial assay

Antibacterial activity of ginger extracts were assessed against Gram-negative (*Escherichia coli, Salmonella typhimurium* and *Pseudomonas aeruginosa*) and Gram-positive (*Staphylococcus aureus, Bacillus subtilis, Listeria monocytogenes*) bacteria strains using the well diffusion method [17]. Dry residue samples were dissolved in 10 % dimethyl sulfoxide (DMSO) to a final concentration of 10 mg/mL. Petridishes were prepared with 15 mL of Muller Hinton agar medium and sterilized by autoclaving at 120 ± 2 °C for 20 min. After inoculation, the Petri dishes were dried for 15 min. Wells of 6 mm diameter were punched off with a sterile Pasteur pipette and filled with ginger extracts (80 μL). The plates were incubated at 37 ± 2 °C foe 24 h. Gentamicin and ciprofloxacin (5 μg/mL) were used as a positive control and 10 % DMSO was used as a negative control. The zone of inhibition that appears after 24 h was measured (in mm) as a property of antibacterial activity of ginger extracts. Test was performed in triplicate.

#### Evaluation of minimum inhibitory concentration (MIC)

The minimum inhibitory concentration (MIC) of ginger extracts was evaluated by micro dilution assay using liquid cultures in 96-well micro plates. A series of diluted extracts were prepared in sterile 96-well micro plates using Mueller Hinton broth. Bacterial suspension (50 μL) was mixed with and equal volume of each dilutions (ranging from 20 to 100 μg/mL). Blank (150 μL broth) and bacteria (100 μL broth and 50 μL bacteria suspantion) were prepared and Gentamicin and ciprofloxacin were used as positive controls. The plates were incubated for 24 h at 37 °C. The diameter of the clear area, directly on the dishes was measured and expressed in millimeter. The MIC was determined by selecting the lowest concentration (highest dilution) of ginger extract that completely showing no growth of the bacteria strains after 24 h. Three replicate plates were used for each concentration.

## Results and discussion

### Dry matter content

Ginger varieties used in this study ranged in dry matter content (DM) from 38.8 % for Halia bentong to 27.2 % for Halia bara (for freshly harvested roots). After 4 months storage, the degree of DM change was temperature-dependent. There was a decline in DM% at 4 months storage at 5 and 15 °C in both the varieties. As shown in Table 1, ginger rhizomes stored for 4 months at 5 °C, had their DM content decreased slightly compared to that of fresh samples; however, at the same storage temperature (5 °C), increasing the storage time to 8 months resulted in a significant decrease in DM content in both the varieties. The decrease in DM content was greater when ginger rhizomes were stored at 15 °C for 8 months. Decrease in the DM content of plant products during storage was reported in previous studies [18]. The extent of storage losses has a direct correlation with several factors including initial moisture content, plant species and parts being stored, storage conditions such as air temperature and movement and relative humidity [19].

**Table 1:** Effect of storage time and temperature on dry matter content, 6-gingerol, 6-shogaol, TPC and TFC in ginger varieties.

	Storage time (month)/temperature	Dry matter (%)	6-gingerol(mg/g DM)	6-shogaol(mg/g DM)	TPC(mg GA/g DM)	TFC(mg Q/g DM)
Halia Bentong	fresh	38.8 ± 2.48^a^	1.47 ± 0.132^d^	1.17 ± 0.04^b^	11.27 ± 1.236^a^	3.79 ± 0.176^c^
	4/(5 °C)	37.0 ± 1.69^a^	1.29 ± 0.127^e^	0.94 ± 0.06^c^	10.73 ± 1.327^ab^	3.58 ± 0.144^c^
	8/(5 °C)	31.0 ± 1.24^c^	0.61 ± 0.122^f^	0.62 ± 0.052^d^	8.21 ± 1.160^c^	3.11 ± 0.129^d^
	4/(15 °C)	33.0 ± 1.19^b^	0.38 ± 0.140^g^	0.44 ± 0.027^e^	6.45 ± 1.066^d^	1.76 ± 0.143^g^
	8/(15 °C)	25.0 ± 1.20^e^	0.30 ± 0.116^g^	0.20 ± 0.016f	5.02 ± 1.128 ^e^	1.19 ± 0.08^h^
Halia Bara	fresh	27.2 ± 1.55^d^	2.73 ± 0.137^a^	1.55 ± 0.058^a^	12.76 ± 1.352^a^	4.28 ± 0.162^a^
	4/(5 °C)	25.2 ± 1.27^e^	2.70 ± 0.119^a^	1.52 ± 0.062^a^	11.62 ± 1.446^a^	4.0 ± 0.177^b^
	8/(5 °C)	20.6 ± 1.12^g^	2.21 ± 0.164^b^	1.16 ± 0.043^b^	8.70 ± 1.088^c^	3.2 ± 0.152^d^
	4/(15 °C)	22.7 ± 1.46^f^	1.79 ± 0.118^c^	0.88 ± 0.033^c^	8.00 ± 1.261^c^	2.46 ± 0.112^e^
	8/(15 °C)	15.1 ± 1.23^h^	1.26 ± 0.127^e^	0.67 ± 0.022^d^	6.80 ± 1.155^d^	2.1 ± 0.094^f^

Data are means of triplicate measurements ± standard deviation. Means not sharing a common single letter in each column for each measurement were significantly different at *p* < 0.05

### Content of 6-gingerol and 6-shogaol

Gingerols are the most pungent component of fresh ginger, contain the following major constituents: 6-gingerol, 8-gingerol, 10-gingerol, and 6-shogaol. As shown in Table 1, 6-gingerol content was influenced by storage temperature, time, and the ginger variety. Halia bara had a higher content of 6-gingerol and 6-shogaol in fresh samples (2.73 and 1.55 mg/g DM) compared to that of Halia bentong (1.47 and 1.17 mg/g DM). After 4 months of storage at 5 °C, there was a slight decrease in 6-gingerol and 6-shogaol content in both the varieties, however, at 8 months of storage at 5 °C, concentration of 6-gingerol and 6-shogaol decreased significantly. At 4 months of storage at a higher temperature of 15 °C, a huge decrease in 6-gingerol (Halia bentong: 70.5 %; Halia bara: 33.7 %) and 6-shogaol content (Halia bentong: 53.19 %; Halia bara: 42.1 %) was observed in both the varieties. Increasing the storage time to 8 months also resulted in significant decrease in the concentrations of 6-gingerol and 6-shogaol compared to those in fresh samples. Storage for 8 months at a temperature of 15 °C decreased 6-gingerol (Halia bentong 50.8 %; Halia bara: 42.9 %) and 6-shogaol content (Halia bentong: 67.7 %; Halia bara: 42.2 %). In summary, the concentrations of 6-gingerol and 6-shogaol decrease significantly with increasing storage time and temperature. After 8 months of storage, the loss of 6-gingerol content was 59.1 and 79.5 %, in Halia bentong and 19.0 and 79.3 % in Halia bara, at 5 °C and 15 °C, respectively, whereas that of 6-shogaol content was 47.0 and 82.9 % in Halia bentong and 25.1 and 56.7 % in Halia bara, at 5 °C, and 15 °C respectively. A recent study, reported a decrease of total gingerol content (83.3 %) in ginger rhizomes during storage at freezing temperature [20].

### Total phenolic and total flavonoid content

TPC and TFC in Halia bentong and Halia bara were also influenced by storage temperature and time (Table 1). Fresh Halia bara rhizome had the highest TPC and TFC (12.76 mg GA/g DM and 4.28 mg Q/g DM) compared to fresh samples of Halia bentong (11.27 mg GA/g DM and 3.79 mg Q/g DM). Storage for 4 months at 5 °C, resulted in a slight decrease in TPC and TFC in these varieties. Storage at the same temperature (5 °C) for a longer duration of 8 months resulted in significant decrease in TPC and TFC contents in both these varieties. Increasing the storage temperature to 15 °C, further diminished the TPC and TFC contents and this decrease continued throughout the 8 months of storage. As shown in Table 1, TPC and TFC in rhizomes stored for 4 months at 15 °C (6.45 mg GA/g DM and 1.76 mg Q/g DM) were lower than those in rhizomes stored for 8 months at 5 °C (8.21 mg GA/g DM and 3.11 mg Q/g DM). This result indicates that storage temperature is more critical than the storage time, for ginger rhizome storage. Overall, during 8 months storage, the loss of TPC was 27.1 % and 55.4 % in Halia bentong, and 31.8 % and 46.7 % in Halia bara, at 5 °C and 15 °C, respectively, whereas that of TFC was 17.9 % and 68.6 % in Halia bentong, and 25.2 % and 50.9 % in Halia bara, at 5 °C, and 15 °C, respectively. A recent publication reported a decrease in the TPC and TFC contents of pistachios (*Pistacia vera* L.) by an increase in the storage temperature [21]. At higher temperatures, there is a change in activity of membrane-bound enzymes and the permeability of the cell membranes [22], which causes an accumulation of toxic intermediatesin the cells [23]. When the plants are stored at a low temperature, the activity of chalcone synthase enzyme (CHS), hydroxycin.namoylquinatetransferase (HQT) and phenylalanine ammonia-lyase (PAL) enzymes (involved in the synthesis of flavonoids and phenolic acids) have been reported to increase considerably [23]. It was expected that the lower storage temperature (5 °C) in this study would have an increased production of the PAL, CHS and HQT enzymes and, subsequently, higher total phenolic and flavonoid content.

### Antioxidant activity

Table 2, shows the DPPH and FRAP activity of the rhizome extracts of ginger varieties stored at different temperatures and for different durations. DPPH radical scavenging activity was used to gauge the antioxidant activity of the ginger extracts from the two varieties. DPPH antioxidant activity in the two varieties and different storage conditions ranged from 16.9 % to 41.7 %. Halia bara showed the highest DPPH activity compared to the Halia bentong rhizome. Highest DPPH activity was observed in fresh rhizomes and rhizomes stored at 5 °C for 4 months with no significant difference observed between the samples. With increasing storage time (from 4 to 8 month) and temperature (from 5 to 15 °C), the DPPH activity of the rhizome extracts decreased significantly. Storage at 5 °C for 8 months decreased the antioxidant activity by 25.8 % and 29.8 % in Halia bentong and Halia bara rhizome extracts, respectively compared to those rhizomes stored at 5 °C for 4 months. Storage at 15 °C for 8 months decreased the antioxidant activity by 34.3 % and 31.5 % in Halia bentong and Halia bara rhizomes extracts respectively compared to those rhizomes stored at 15°C for 4 months. As shown in the results in Table 2, antioxidant activity was affected more by storage temperature than the duration of storage. A significant decrease in DPPH activity was observed when storage temperature was raised from 5 to 15 °C. Halia bentong and Halia bara rhizomes extracts showed lowest DPPH activity compared to positive controls (Gallic acid: 76.1 % and quercetin: 82.4 %).

**Table 2:** Effect of storage time and temperature on antioxidant activities and IC_50_ value of ginger varieties

	Storage time (month)/temperature	DPPH (%)	FRAP (μM of Fe (II)/g DM)	IC_50_ (DPPH)
Halia Bentong	fresh	41.7 ± 1.33^b^	488.3 ± 29.56^b^	39.2 ± 1.50^f^
	4/(5 °C)	41.0 ± 1.49^b^	462.8 ± 24.33^b^	39.3 ± 1.22^f^
	8/(5 °C)	30.4 ± 1.58^d^	371.2 ± 28.16^e^	42.4 ± 1.29^e^
	4/(15 °C)	22.4 ± 1.04^f^	266.7 ± 18.76^f^	51.4 ± 1.42^c^
	8/(15 °C)	14.7 ± 0.44^h^	176.1 ± 14.29^g^	66.7 ± 1.19^a^
Halia Bara	fresh	49.7 ± 1.88^a^	537.4 ± 28.76^a^	29.4 ± 1.59^h^
	4/(5 °C)	47.6 ± 1.29^a^	511.3 ± 32.16^a^	29.8 ± 1.26^h^
	8/(5 °C)	33.4 ± 1.16^c^	422.1 ± 24.38^c^	33.4 ± 1.13^g^
	4/(15 °C)	24.7 ± 1.77^e^	327.6 ± 20.19^d^	46.7 ± 1.75^d^
	8/(15 °C)	16.9 ± 0.93^g^	244.5 ± 18.46^f^	55.2 ± 1.06^b^
Gallic acid		76.1 ± 2.16	644.5 ± 30.52	24.6 ± 1.05
Quercetin		82.4 ± 2.44	729.2 ± 32.16	15.9 ± 1.27

Data are means of triplicate measurements ± standard deviation. Means not sharing a common single letter in each column for each measurement were significantly different at *p* < 0.05

The FRAP value of rhizome extracts ranged from 244.5–537.4 μM of Fe (II)/g. The highest FRAP activity was observed in the fresh rhizomes of Halia bara. No significant differences between fresh samples and samples stored for 4 month at 5 °C were observed. Results of the FRAP assay showed that increasing the storage time and temperature reduced the FRAP activity of ginger varieties significantly. Largest decrease was observed when samples were stored for 8 month at 15 °C.

IC_50_ value (half maximal inhibitory concentration) indicates the free radical inhibitory strength (low IC_50_ values mean stronger free radical inhibition at low concentrations). As observed from the IC_50_ results, lowest IC_50_ value was observed in fresh samples followed by samples stored for 4 months at 5 °C. This may be explained by the instability of compounds that lowered the DPPH activity with an increase in storage time and temperature. Another reason for such a trend may be the variations in the interaction between the inhibitors present and the reagents at different storage temperatures and durations. IC_50_ values of ginger varieties were higher than that of the positive controls (Gallic acid: 24.6 μg/mL and quercetin: 15.9 μg/mL). The decrease in antioxidant potential in the rhizomes with longer storage times and higher temperatures could be related to the reduction in the content of some phytochemicals, such as gingerols, phenolic acids and, flavonoids. Positive correlation between level of secondary metabolites and antioxidant properties was demonstrated by previous studies [24–26]. Our finding is consistent with previous studies indicating that the levels of secondary metabolites (phenolics and flavonoids) correspond to the free radical scavenging potential of the medicinal plants [1, 17, 25].

### Identification and separation of flavonoids and phenolic acids

In this study, five flavonoids (quercetin, rutin, catechin, epicatechin, and kaempferol) and four phenolic acids (gallic acid, ferulic acid, cinnamic acid, and tannic acid) were identified in Halia bentong and Halia bara rhizome extracts stored for different durations and at different temperatures (Table 3). The results showed that with increasing storage time and temperature, concentrations of the identified compounds decreased significantly in those varieties. On an average, Halia bara showed higher concentration of phytochemicals compared to Halia bentong.

**Table 3:** Effect of storage time and temperature on individual flavonoids and phenolic acids content in rhizome extracts of ginger varieties

Ginger varieties	Storage time (month)/temperature	Quercetin	Rutin	Catechin	Epicatechin	Kaempferol	Gallic acid	Ferulic acid	Cinnamic acid	Tannic acid
Halia Bentong	fresh	0.922 ± 0.113^b^	0.437 ± 0.034^a^	0.422 ± 0.027^c^	0.135 ± 0.016^c^	0.076 ± 0.014^d^	0.178 ± 0.056^b^	0.122 ± 0.018^b^	0.048 ± 0.016^b^	0.077 ± 0.011^b^
	4/(5 °C)	0.916 ± 0.124^b^	0.430 ± 0.029^a^	0.402 ± 0.034^c^	0.133 ± 0.018^c^	0.075 ± 0.016^d^	0.172 ± 0.049^b^	0.116 ± 0.016^b^	0.04 ± 0.016^b^	0.068 ± 0.009^b^
	8/(5 °C)	0.729 ± 0.105^c^	0.311 ± 0.041^b^	0.273 ± 0.022^e^	0.100 ± 0.022^d^	0.044 ± 0.014^d^	0.113 ± 0.021^c^	0.088 ± 0.015^c^	0.02 ± 0.010^c^	0.031 ± 0.008^d^
	4/(15 °C)	0.511 ± 0.096^d^	0.168 ± 0.015^c^	0.127 ± 0.019^f^	ND	ND	0.100 ± 0.018^c^	0.042 ± 0.014^d^	ND	ND
	8/(15 °C)	0.50 ± 0.088^d^	0.120 ± 0.011^d^	0.056 ± 0.013^g^	ND	ND	0.061 ± 0.011^d^	ND	ND	ND
Halia Bara	fresh	1.160 ± 0.126^a^	0.355 ± 0.032^b^	0.568 ± 0.059^a^	0.166 ± 0.063^a^	0.96 ± 0.124^a^	0.211 ± 0.042^a^	0.162 ± 0.063^a^	0.091 ± 0.026^a^	0.122 ± 0.019^a^
	4/(5 °C)	1.100 ± 0.137^a^	0.322 ± 0.027^b^	0.517 ± 0.063^a^	0.144 ± 0.012^b^	0.86 ± 0.116^a^	0.210 ± 0.033^a^	0.146 ± 0.032^a^	0.083 ± 0.016^a^	0.120 ± 0.016^a^
	8/(5 °C)	0.762 ± 0.092^c^	0.300 ± 0.042^b^	0.488 ± 0.034^b^	0.122 ± 0.017^c^	0.80 ± 0.114^a^	0.155 ± 0.024^b^	0.121 ± 0.017^b^	0.043 ± 0.031^b^	0.110 ± 0.013^a^
	4/(15 °C)	0.426 ± 0.076^e^	0.156 ± 0.018^c^	0.416 ± 0.024^c^	0.110 ± 0.016^d^	0.619 ± 0.076^b^	0.110 ± 0.019^c^	0.066 ± 0.051^c^	ND	ND
	8/(15 °C)	0.281 ± 0.049^f^	0.112 ± 0.021^d^	0.362 ± 0.030^d^	0.064 ± 0.018^e^	0.522 ± 0.053^c^	0.056 ± 0.014^d^	0.029 ± 0.010^e^	ND	ND

Data are means of triplicate measurements ± standard deviation. Means not sharing a common single letter in each column for each measurement were significantly different at *p* < 0.05. The units of all measurement are mg/g DM

*ND* not detected

Epicatechin, kaempferol, cinnamic acid, and tannic acid were not detected in Halia bentong rhizome samples stored at 15 °C. Ferulic acid was not detected in Halia bentong rhizomes stored at 15 °C for 8 months. Cinnamic acid was not detected in extracts of Halia bara rhizomes stored at 15 °C for 8 months. Tannic acid was not detected in the rhizome extracts of both the varieties stored at 15 °C for 8 months. Among the identified phenolic acids, cinnamic acid and tannic acid were not detected when the storage temperature was raised from 5 to 15 °C, indicating that they were more sensitive to temperature than other detected phenolic acids. The flavonoid compounds, epicatechin and kaempferol also showed the same trend but only in Halia bentong samples. HPLC analysis for individual flavonoids and phenolic acids indicated that quercetin and gallic acid, respectively, were the major compounds in both Halia bentong and Halia bara rhizomes extracts. The order of abundance of the flavonoids identified in this study is as follows: quercetin > catechin > rutin > kaempferol > epicatechin. The order of abundance of the phenolic acids identified in this study is as follows: gallic acid > ferulic acid > tannic acid > cinnamic acid. The decrease in flavonoid and phenolic component concentrations during storage at 15 °C, can be attributed to the degradation of polyphenols by an active enzyme system in the tissue including chalcone synthase [1], polyphenol oxidase and peroxidase [27].

### Polyphenol oxidase activity

**Fig. 1:**
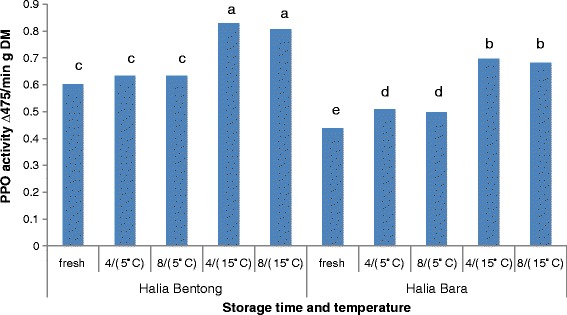
Effect of different storage time and temperature on polyphenol oxidase activityin ginger rhizomes. Means not sharing a common single letter were significantly different at *p* < 0.05

Polyphenol stability under different conditions is a very important aspect that must be taken into account to ensure that phenolic compounds possess the desired properties and maintain their activity under different storage conditions, which can involve high temperatures and light. Figure 1 shows the results of PPO activity in rhizomes stored at different conditions. Significant differences were observed between storage temperature and time for PPO activity. Highest PPO activity was observed in Halia bentong rhizomes stored at 15 °C for 4 and 8 months respectively followed by Halia bara rhizomes stored at 15 °C for 4 and 8 months respectively. In the present study, fresh samples represent the lowest PPO activity in those varieties with highest content of phytochemicals and antioxidant activity compared to stored samples. This could be due to activation of deteriorative enzymes such as lipoxygenase and PPO during storage process. PPO, mixtures of monophenol oxidase, and catechol oxidase enzymes are widely present in plant tissues and can oxidise diphenols in the presence of oxygen molecules, causing rapid enzymatic oxidation of natural antioxidants. Lower TPC and TFC in stored samples are probably due to the oxidation by PPO. Oxidation of quercetin by PPO has beenreported previously [28].

### Antibacterial activity

The antibacterial activity of Halia bara and Halia bentong rhizome extracts, stored at different temperatures for different durations, against both Gram-negative and Gram-positive bacteria are shown in Table 4. Rhizome extracts of both the ginger varieties demonstrated valuable antibacterial activity against Gram-negative and Gram-positive bacterial strains. Except against *Escherichia coli, Salmonella typhimurium* and *Pseudomonas aeruginosa*, extracts of stored Halia bara rhizome showed higher antibacterial activity than that of the Halia bentong extracts. It is clear that there is a relationship between the high antibacterial activity of the Halia barar hizomes and the presence of flavonoids and phenolic components, such as quercetin and gallic acid. However, there was a variation in the size of the inhibition zones among the different groups of bacteria. Fresh rhizome samples of both the varieties showed potent antibacterial activity against bacterial strains compared to stored rhizome extracts. Increasing the storage temperature from 5 to 15 °C and storage time from 4 to 8 months significantly reduced the antibacterial activity in both the varieties. Notably, fresh rhizomes and rhizomes stored for 4 months at 5 °C exhibited higher antibacterial activity against *Staphylococcus aureus* compared to positive controls (gentamicin and ciprofloxacin). Rhizomes stored at 15 °C for 4 and 8 months did not show any antibacterial activity against Gram-negative bacteria. The reason that Gram-positive bacteria have a higher antibacterial-sensitivity than Gram-negative bacteria could be the differences in their cell membrane constituents. The outer membrane of the Gram-negative cell wall comprises structural lipopolysaccharides, which render the cell wall impermeable to lipophilic solutes, unlike Gram-positive bacteria, which lack this outer membrane. This morphological difference influences their sensitivity to antibacterial agents. Table 5 shows the minimum inhibitory concentration (MIC) of Halia bentong and Halia bara rhizome extracts stored at different temperatures and for different durations, against the tested pathogenic organisms. The MIC of the extract varied from 40 to > 100 μg/mL for Gram-positive bacteria, and 60 to > 100 μg/mL for Gram-negative bacteria. Most Zingiberaceae plant extracts exhibit antimicrobial activity [29–31]. The rhizome extracts of ginger have previously demonstrated antibacterial activity against *Escherichia coli, Staphylococcus aureus, Bacillus subtilis,* and *Pseudomonas aeruginosa*, with MIC of > 20, 0.0024, 0.625, and 2.5 mg/mL, respectively [32]. Antibacterial activity of gingerol compounds (6-gingerol, 8- gingerol, and 10-gingerol) isolated from ginger rhizomes was reported previously [33]. In particular, it was reported that antimicrobial, antifungal and pharmaceutical propertiesof ginger is related to shogaols and gingerols [34]. The growing concern about health problems in the recent times is leading to the development of natural antimicrobials to control microbial diseases. Medicinal plants and spices are commonly used natural antimicrobial agents and have been used traditionally for thousands of years by many cultures to tackle common health problems. Drug discovery based on naturally-occurring plant-derived antimicrobials, attained paramount importance because these new drugs are likely to be effective against multi-drug resistant microbes [35]. The bacterial properties of herbs is believed to be due to phytochemicals and secondary metabolites such as flavonoids and phenolics [36, 37]. In current study, increasing the storage temperature from 5 to 15 °C significantly decreased the antibacterial activity against Gram-positive bacterial strains and completely abolished the effectiveness against Gram-negative bacterial strains. A possible explanation for this might be that proper storage at 15 °C helped to halt the degradation of phytochemicals. Previous studies have confirmed that natural antioxidants play a significant role in the prevention and treatment of metabolic disorders [38–40]. Therefore, the possibility of using simple methods to keep or even increase phytochemical content in herbs, to enhance the bioactivities simultaneously, is worthy of further investigation. In current study antibacterial activity was tested on available bacteria strains and it can be mentioned as a limitation of this study. Also we did not test antifungal activity of stored ginger varieties. Then, for future studies we suggest to test effect of storage temperature and time of ginger on varied range of bacteria and fungus strains.

**Table 4:** Effect of storage time and temperature on antibacterial activity of rhizome extracts against bacterial strains

Ginger varieties		Inhibition zone (mm)					
	Storage time (month)/temperature	*S. aureus*	*B.subtilis*	*L.monocytogenes*	*E. coli*	*S. typhimurium*	*P. aeruginosa*
Halia Bentong	fresh	11.5 ± 1.33^b^	12.5 ± 1.35^c^	14.0 ± 1.66^b^	12.0 ± 1.25^b^	11.0 ± 1.27^a^	13.5 ± 1.28^a^
	4/(5 °C)	8.5 ± 0.69^b^	7.1 ± 0.68^b^	9.0 ± 0.73^c^	8.6 ± 0.54^c^	8.3 ± 0.26^b^	9.5 ± 1.01^b^
	8/(5 °C)	5.3 ± 0.373^d^	4.2 ± 0.67^d^	5.6 ± 0.144^e^	3.5 ± 0.21^e^	3.5 ± 0.19^d^	5.6 ± 0.19^d^
	4/(15 °C)	3.1 ± 0.22^f^	2.2 ± 0.12^e^	5.5 ± 0.16^e^	NO	NO	NO
	8/(15 °C)	1.4 ± 0.13^g^	1.2 ± 0.08^g^	2.2 ± 0.09^g^	NO	NO	NO
Halia Bara	fresh	14.0 ± 2.62^a^	15.5 ± 2.52^a^	16.0 ± 2.15^a^	13.5 ± 1.55^a^	12.0 ± 1.42^b^	15.0 ± 1.26^b^
	4/(5 °C)	9.2 ± 0.58^b^	8.5 ± 0.47^a^	11.5 ± 1. 88^b^	6.8 ± 0.46^c^	7.8 ± 0.81^c^	8.0 ± 0.61^c^
	8/(5 °C)	6.4 ± 0.467^c^	4.6 ± 0.33^c^	6.5 ± 0.72^e^	2.0 ± 0.17^d^	2.4 ± 0.28^d^	3.5 ± 0.27^d^
	4/(15 °C)	4.2 ± 0.329^e^	3.6 ± 0.29^d^	7.5 ± 0.58^d^	NO	NO	NO
	8/(15 °C)	2.2 ± 0.149^g^	2.8 ± 0.18^f^	3.40 ± 0.15^f^	NO	NO	NO

Data are means of triplicate measurements ± standard deviation. Means not sharing a common single letter in each column for each measurement were significantly different at *p* < 0.05. *NO* not obsorved

**Table 5:** Effect of storage time and temperature on minimum inhibition concentration (MIC) of rhizome extracts against bacterial strains.

Ginger varieties	Storage time (month)/temperature	*S. aureus*	*B.subtilis*	*L.monocytogenes*	*E. coli*	*S. typhimurium*	*P. aeruginosa*
Halia Bentong	fresh	40	60	80	80	60	60
	4/(5 °C)	40	60	80	>100	>100	80
	8/(5 °C)	60	80	>100	>100	>100	>100
	4/(15 °C)	>100	>100	>100	NO	NO	NO
	8/(15 °C)	>100	>100	>100	NO	NO	NO
Halia Bara	fresh	40	60	80	80	60	60
	4/(5 °C)	40	60	80	80	80	80
	8/(5 °C)	60	80	>100	>100	>100	>100
	4/(15 °C)	80	>100	>100	NO	NO	NO
	8/(15 °C)	>100	>100	>100	NO	NO	NO

Data are means of triplicate measurements . *NO* not obsorved. Unit: μg/mL

## Conclusions

The results of this study indicate that storage temperature and time significantly affect ginger phytochemical content, antioxidant and antibacterial properties, and overall quality. 6-gingerol, 6-shogaol, TPC, TFC, and individual phenolic acids and flavonoids were present in higher concentrations in Halia bara compared to Halia bentong. The free radical scavenging activity was affected more by storage temperature than by storage time. The overall quality of the rhizomes was best when stored at low temperature (5 °C) and decreased as the storage time was extended. Highest and lowest activity of PPO was observed in stored and fresh rhizomes respectively, and lower TPC and TFC in stored samples are probably due to the oxidation by PPO during storage time. Fresh rhizomes and those stored at 5 °C for 4 months exhibited acceptable levels of antibacterial activity against both Gram-positive and Gram-negative bacterial strains. From these results, it seems that fresh rhizome quality could be maintained for up to 4 months when stored at 5 °C. Future research should focus on determining which of the components shown in Table 4 can be used as a quality index to determine the optimal storage conditions for ginger rhizomes.

## Abbreviations

DMSO: Dimethyl sulfoxide
DPPH: 1,1-diphenyl-2-picrylhydrazyl
FRAP: Ferric reducing antioxidant potential
MIC: Minimum inhibitory concentration
TFC: Total flavonoid content
TPC: Total phenolic content
UHPLC: Ultra-high performance liquid chromatography
